# HIV infection during pregnancy in the state of Rio de Janeiro, Brazil, 2021-2023

**DOI:** 10.1590/1980-549720250020

**Published:** 2025-05-09

**Authors:** Rosa Maria Soares Madeira Domingues, Marcos Augusto Bastos Dias, Ana Paula Esteves-Pereira, Paula Mendes Luz, Emilia Jalil, Vania Rocha, Angela Cristina Vasconcelos de Andrade Rabello, Ruth Khalili Friedman, Maria do Carmo Leal

**Affiliations:** I Fundação Oswaldo Cruz, Evandro Chagas National Institute of Infectious Diseases – Rio de Janeiro (RJ), Brazil.; II Fundação Oswaldo Cruz, Fernandes Figueira National Institute for Women, Children and Adolescents’ Health – Rio de Janeiro (RJ), Brazil.; III Fundação Oswaldo Cruz, Sérgio Arouca National School of Public Health – Rio de Janeiro (RJ), Brazil.

**Keywords:** Pregnancy, HIV Infections, Prenatal Care, Health Evaluation

## Abstract

**Objectives:**

To estimate the prevalence of HIV infection in pregnant women; analyze the gestational and maternal outcomes of women with HIV; and evaluate process indicators for the prevention of vertical transmission of HIV according to type of financing for hospital admission in the state of Rio de Janeiro (RJ).

**Methods:**

cross-sectional study with 1,923 women, conducted between 2021-2023. Interviews were carried out with women, and data was extracted from the pregnancy booklet and hospital records. The prevalence of HIV infection, gestational and maternal outcomes, and the adequacy of process indicators for the management of HIV infection were estimated with respective 95% confidence intervals (95%CI) according to the type of financing — public or private — for hospital admission for childbirth or abortion.

**Results:**

Coverage of prenatal care (PNC), HIV testing during PNC (one and two tests), and testing during hospital admission was 93.7, 79.7, 45.8, and 79.2%, respectively. The prevalence of HIV infection was estimated at 0.79% (95%CI 0.31–1.99). Only 40% of women with HIV had registered antiretroviral treatment and 26% had registered viral load tests in their pregnancy booklet. Women with public funding were more socially vulnerable and had less coverage of PNC and testing with two tests.

**Conclusion:**

Missed opportunities were identified in the management of pregnant women with HIV in public and private services in RJ. The detection rate was higher than that of the Notifiable Diseases Information System and suggests underreporting of cases.

## INTRODUCTION

Vertical transmission (VT) of the human immunodeficiency virus (HIV), which occurs from mother to child during pregnancy, childbirth, or breastfeeding, is the primary mode of HIV transmission in children. This transmission is preventable with the available care resources^1^. However, gaps in access to services and their integration undermine efforts to meet global objectives for the elimination of VT of HIV^2,3^. In Brazil, resources and guidelines for managing HIV during pregnancy have been available since the 1990s. Despite this, challenges persist in the implementation of these measures, resulting in missed opportunities for VT prevention^4–6^.

In 2022, the HIV detection rate among pregnant women in Brazil was 3.1 per 1,000 live births (LB), while the AIDS detection rate in children under 5 years of age, which is used for VT monitoring, was 1.5 cases/100,000 inhabitants. In the same year, the state of Rio de Janeiro had the third highest national rate of HIV detection in pregnant women (5.1 per 1,000 LB) and the third highest rate of AIDS detection in children under 5 years of age (3.8 per 100,000 inhabitants)^7^.

Previous studies conducted in the city of Rio de Janeiro and the metropolitan region of the state have highlighted deficiencies in HIV testing among pregnant women during prenatal care (PN)^8^ and in the PN, perinatal, and postnatal care cascade^9^.

This study aimed to:

- estimate the prevalence of HIV infection in pregnant women;
- analyze the gestational and maternal outcomes of women with HIV infection and children exposed to HIV;
- evaluate some process indicators for preventing VT, according to the type of financing for hospital admissions for childbirth or abortion in RJ, using data from the *Nascer no Brasil II* survey.

## METHODS

This study is a part of the research project "Birth in Brazil II: National Research on Abortion, Labor and Childbirth" (*Nascer no Brasil II: pesquisa nacional sobre aborto, parto e nascimento* – NBII).

### The NBII research

This was a cross-sectional, hospital-based, nationwide study. A two-stage probabilistic sampling method was employed, involving the selection of hospitals and women with their newborns. Hospitals were stratified by macroregion, location (metropolitan or non-metropolitan area), type (public, mixed, private), and size (100-499, ≥500 LB/year). Thirty postpartum women were interviewed at hospitals with 100-499 LB/year, while 50 women were interviewed at hospitals with ≥500 LB/year.

Women who had recently given birth to a newborn of any weight or gestational age (GA), women who had experienced stillbirths with a GA ≥22 weeks or weight ≥500 g, and women hospitalized with a diagnosis of miscarriage were eligible for inclusion. Women who had given birth outside of a hospital, delivered triplets or more, had communication difficulties, were discharged while still pregnant, or were hospitalized for delivery by court order were excluded from the study.

Interviews were conducted in the immediate postpartum period, and data were extracted from the pregnant woman's booklets and hospital records. The protocol for the NBII study is published in Leal et al.^10^.

### Study of the state of Rio de Janeiro

The sample of postpartum women in the state of Rio de Janeiro was calculated using the same parameters as the national study: a cesarean section rate of 57% (in RJ, 2019), a significance level of 5%, and a power of 90% to detect differences of 7%. A design effect of 1.3 was applied, resulting in a minimum sample size of 1,350 women. To achieve this number, the sample size was increased from 50 to 90 postpartum women in public and mixed hospitals with ≥500 LB/year (Suppl. Mat. 1: https://doi.org/10.7303/syn64828293.1). Hospital interviews were conducted between November 2021 and June 2023 at 29 hospitals across 18 municipalities (13 public in 12 municipalities, three mixed in three municipalities, and 13 private in six municipalities).

### Variables and data source

The following indicators were analyzed based on recommendations from the World Health Organization (WHO)^1,11,12^ and the Brazilian Ministry of Health^13^:

- PN Assistance: having received at least one PN consultation^1^;
- Receipt of the pregnancy booklet (PB) during PN assistance^11^;
- Early initiation: first PN consultation up to the 12^th^ week of pregnancy^11^;
- Adequacy of the number of PN consultations: number of consultations for GA at delivery, considering the calendar and the minimum number of eight consultations recommended by the WHO^11^;
- HIV testing during pregnancy: first testing at the beginning of PN care and second testing from the 28^th^ week of pregnancy onward^13^, analyzed only in women with GA≥34 weeks at the time of delivery;
- HIV testing upon hospital admission for childbirth or abortion assistance^13^;
- Prevalence of HIV infection during pregnancy and detection rate per 1,000 LB: HIV infection defined by a diagnosis record prior to the current pregnancy and/or testing during PN care and/or hospital admission with a positive result and/or diagnosis of HIV infection during pregnancy, including records of diagnosis during pregnancy, cause of hospitalization, indication for cesarean section or diagnosis of HIV-exposed child. Cases were classified based on the timing of the diagnosis (prior to PN care, during PN care, at hospital admission). The prevalence of HIV infection was calculated considering all pregnancies in the denominator, while the detection rate was calculated in relation to the number of LB^7^;
- HIV viral load testing during PN care: proportion of pregnant women with HIV who had a recorded viral load test result during PN care^13^;
- Treatment with antiretroviral therapy (ART) during pregnancy: proportion of pregnant women with HIV with a recorded prescription of ART during pregnancy^11,13^;
- Mode of delivery: proportion of women with HIV who had vaginal delivery, cesarean section without labor, and cesarean section with labor;
- Maternal outcome: proportion of pregnant women with HIV classified as cases of potentially life-threatening conditions, maternal near miss, and maternal death, according to WHO definitions^12^;
- Pregnancy outcome: proportion of the outcomes "early fetal loss" (fetal death with weight <500 g and GA <22 weeks), "stillbirth" (fetal death with weight ≥500 g or GA ≥22 weeks), "neonatal death" (death occurring up to the 27^th^ day of life), prematurity (LB with less than 37 gestational weeks), and low birth weight (LB with birth weight <2,500 g) in women with HIV during pregnancy.

The following maternal characteristics were analyzed: "type of hospitalization financing" ("public:" for women treated in a public hospital or a private hospital with hospitalization covered by the Unified Health System; "private:" for women treated in a private hospital with hospitalization covered by health insurance or out-of-pocket payment); age (<20, 20 to 34, ≥35 years); race/skin color (white, brown, black); years of completed education (≤8, 9 to 11, 12 to 15, ≥16 years); and marital status (living with a partner or not).

Data on maternal characteristics, receipt of PB, timing of PN initiation, and the number of consultations were obtained through hospital interviews with the women. Information on blood tests and the prescription of ART conducted during PN was extracted from the PB, while data on blood tests performed during hospital admission and pregnancy outcomes were obtained from hospital records. Blood tests and medications not documented in either the PB or hospital records were considered not performed.

### Data analysis

All analyses were conducted for the total sample and stratified by type of hospitalization financing. The full sample was used to describe maternal characteristics and estimate the prevalence of HIV infection, HIV testing during hospitalization, and maternal and gestational outcomes. However, for the analysis of indicators related to tests and medications used during PN care, women without their PB recorded were excluded.

Initially, the demographic and social characteristics of the women were analyzed. Subsequently, indicators of HIV infection management during pregnancy were estimated, along with their respective 95% confidence intervals (95%CI). The reference standard of ≥95%, as recommended by the WHO^1^ for process indicators in the prevention of HIV VT, was adopted.

The analyses were conducted using IBM Statistical Package for the Social Sciences Statistics for Windows, Version 19.0 (IBM Corp., Armonk, NY, USA), incorporating design effect, weighting, and sample calibration.

The NBII research was approved by the National Research Ethics Commission, Certificate of Presentation of Ethical Appreciation: 21633519.5.0000.5240, on June 22, 2020, and received approval from the local research ethics committees of the institutions or the clinical board in the absence of local committees.

## RESULTS

Less than 5% of women were ineligible, refused to participate, or were lost due to hospital discharge before the interview, and these women were replaced at the same hospital. A total of 1,923 women were interviewed, of whom 91.6% (1,762/1,923) were hospitalized for childbirth and 8.4% (161/1,923) were hospitalized due to a diagnosis of abortion. Approximately one-quarter of the women had private financing for hospital care (477/1,923). Most women were between 20 and 34 years old, identified as brown, had 12 or more years of education, and lived with a partner. PN care coverage was 93.7% (1,802/1,923), with a significantly lower proportion among women hospitalized due to abortion (41.8%, 67/161). Among women who received PN care, 98.1% (1,767/1,802) received PB, 77.8% (1,401/1,802) initiated PN care early, and 71.5% (1,289/1,802) had an adequate number of consultations. Women with public funding had a higher proportion of adolescents (age <20 years), brown and black women, and those with up to 11 years of education. In contrast, women with private funding had a higher proportion of those aged 35 years old or older, white women, women with 16 or more years of education, and those living with a partner. Women with private funding had greater PN care coverage, a higher proportion of early initiation and adequate consultation numbers, but a lower proportion of women receiving PB (Table 1).

**Table 1:** Social, demographic, and prenatal care utilization characteristics by type of hospital admission funding. State of Rio de Janeiro, 2021–2023.

Characteristics		Total (N=1,923) n (%: 95%CI)	Public funding (N=1,446) n (%: 95%CI)	Private funding (N=477) n (%: 95%CI)
**Type of hospital admission**				
	Postpartum	1,762 (91.6: 88.9–93.7)	1,305 (90.2: 87.8–92.2)	457 (95.8: 86.3-–98.8)
	Abortion	161 (8.4: 6.3–11.1)	141 (9.8: 7.8–12.2)	20 (4.2: 1.2–13.7)
**Maternal age (years)**				
	<20	188 (9.7: 7.8–12.2)	181 (12.5: 10.7–14.5)	7 (1.4: 0.5–3.8)
	20 to 34	1,362 (70.8: 68.5–73.1)	1,055 (73.0: 69.8–75.9)	307 (64.4: 59.3–69.1)
	35 and older	373 (19.4: 17.2–21.8)	210 (14.5: 12.9–16.4)	163 (34.2: 29.1–39.7)
**Skin color**				
	White	569 (29.6: 25.0–34.6)	331 (22.9: 18.6–27.8)	238 (50.0: 43.8–56.2)
	Black	390 (20.3: 16.7–24.4)	346 (23.9: 20.8–27.4)	44 (9.2: 6.5–12.9)
	Brown	964 (50.1: 46.7–53.5)	769 (53.2: 48.7–57.6)	195 (40.8: 34.0–48.0)
**Years of schooling**				
	≤8	290 (15.1: 12.5–18.1)	286 (19.8: 17.2–22.6)	4 (0.9: 0.5–1.6)
	9 to 11	538 (28.0: 21.2–35.9)	499 (34.5: 28.5–41.1)	39 (8.1: 4.8–13.4)
	12 to 15	789 (41.0: 35.5–46.8)	586 (40.5: 33.7–47.7)	203 (42.5: 34.6–50.9)
	16 or more	306 (15.9: 11.3–21.8)	75 (5.2: 3.6–7.2)	231 (48.4: 37.3–59.6)
**Lives with a partner**		1,535 (79.9: 75.6–83.6)	1,089 (75.4: 71.8–78.7)	446 (93.5: 89.6–96.0)
**Had prenatal care (one or more consultations)**		1,802 (93.7: 91.8–95.2)	1,332 (92.1: 90.0–93.8)	470 (98.5: 95.9–99.4)
	Postpartum women (total = 1,762, public = 1,305, private = 457)	1,735 (98.5: 97.5–99.1)	1,278 (98.0: 96.5–98.8)	456 (99.9: 98.9–100)
	Post-abortion women (total = 161, public = 141, private = 20)	67 (41.8: 32.7–51.4)	54 (38.3: 30.2–47.2)	13 (66.3: 53.8–76.9)
**Women with prenatal care**		**(N=1,802)** **n (%: IC95%)**	**(N=1,332)** **n (%: IC95%)**	**(N=470)** **n (%: IC95%)**
**Received pregnancy booklet**		1,767 (98.1: 96.3–99.0)	1,325 (99.5: 98.1–99.8)	442 (94.2: 90.0–96.6)
**Early initiation (up to 12 weeks)**		1,401 (77.8: 74.7–80.5)	963 (72.3: 69.0–75.3)	438 (93.3: 90.8–95.2)
**Adequate number of consultations**		1,289 (71.5: 68.4–74.5)	898 (67.4: 63.3–71.3)	391 (83.2: 76.7–88.1)

95%: 95% confidence interval.

**Figure 1:**
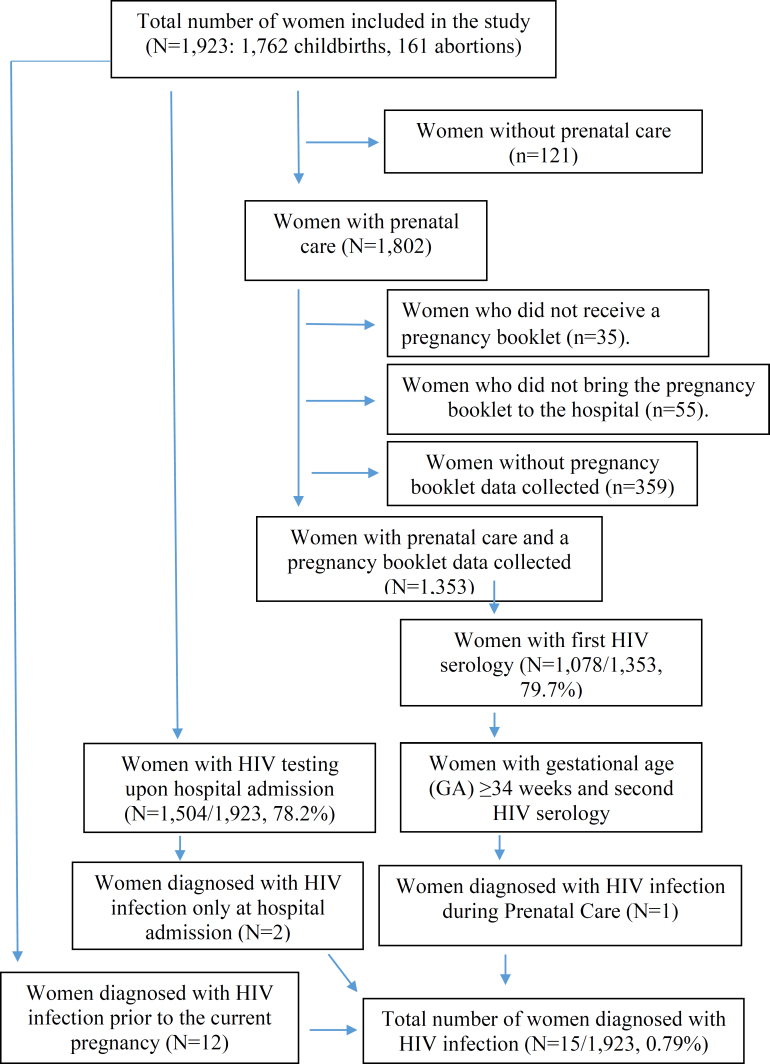
Flowchart of women analyzed in the study and estimation of pregnant women diagnosed with the Human Immunodeficiency Virus (HIV). State of Rio de Janeiro, 2021–2023.

Of the total number of interviewees, 70.3% (1,353/1,923) had their PB data collected (Figure 1), with a lower proportion among women with private financing (60.9% [255/418] private *vs*. 84.8% [1,098/1,295] public, p<0.001). Among the women with PB data collected, 79.7% (1,078/1,353) and 45.8% (595/1,298) had their first and second HIV tests performed during the PN period, respectively. At the time of hospital admission, HIV testing was recorded in 78.2% (1,504/1,923) of the medical records, with no significant difference between hospitalizations for childbirth or abortion care. Higher coverage of the second HIV test during the PN period was observed in women with private financing, while higher coverage of testing at hospital admission was observed in women with public financing. This trend was consistent for both hospital admissions for childbirth (97.7% [1,274/1,305] public, 20.7% [95/457] private) and for abortion care (93.3% [132/141] public, 17.7% [3/20] private) (Table 2).

**Table 2:** Prevalence of Human Immunodeficiency Virus (HIV) infection during pregnancy and HIV infection management indicators, according to the type of hospitalization financing. State of Rio de Janeiro, 2021–2023*.

Indicators		Total	Public funding	Private funding
**Women with the Pregnancy Booklet**		**N=1,353** **n (%: 95%CI)**	**N=1,098** **n (%: 95%CI)**	**N=255** **n (%: 95%CI)**
First HIV test during prenatal care		1,078 (79.7: 73.5–84.7)	887 (80.7%: 73.5–86.3)	191 (75.1: 69.0–80.4)
**Women with the Pregnancy Booklet and gestational age at delivery ≥34 weeks**		N=1,298 n (%: 95%CI)	N=1,047 n (%: 95%CI)	N=251 n (%: 95%CI)
Second HIV test during prenatal care		595 (45.8: 39.0–52.7)	452 (43.2: 34.9–51.9)	143 (56.8: 47.1–66.0)
**Total women**		**(N=1,923)** **n (%: 95%CI)**	**(N=1,446)** **n (%: 95%CI)**	**(N=477)** **n (%: 95%CI)**
**HIV test during hospitalization (postpartum or abortion)**		1,504 (78.2: 67.2–86.3)	1,406 (97.2: 93.0–98.9)	98 (20.6: 14.2–28.8)
	Postpartum (total=1,762, public=1,305, private=457)	1,369 (77.7: 66.7–85.8)	1,274 (97.7: 93.1–99.2)	95 (20.7: 14.1–29.3)
	Abortion (total=161, public=141, private=20)	135 (83.7: 65.1–93.4)	132 (93.0: 80.3–97.8)	3 (17.7: 5.9–42.8)
**HIV infection prevalence**		15 (0.79: 0.31–1.99)	14 (0.96: 0.33–2.73)	1 (0.27: 0.05–1.50)
	Women admitted for childbirth care (total=1,762, public=1,305, private=457)	14 (0.82: 0.32–2.08)	13 (1.01: 0.35–2.90)	1 (0.28: 0.05–1.54)
	Women admitted for abortion care (total=161, public=141, private=20)	1 (0.39: 0.05–3.13)	1 (0.45: 0.05–3.60)	0
	Detection rate per 1,000 live births (total=1,752, public=1,295, private=457)	15 (7.89: 3.14–19.70)	14 (9.68: 3.38–27.42)	1 (2.81:0.51–15.4)
**Women diagnosed with HIV**		**N=15** **n (%: 95%CI)**	**N=14** **n (%: 95%CI)**	**N=1** **n (%: 95%CI)**
**Timing of diagnosis**				
	Before the current pregnancy	12 (79.7: 18.9–98.5)	11 (77.8: 16.6–98.4)	1 (100: 100–100)
	During prenatal care	1 (7.6:0.7–48.4)	1 (8.3: 0.8–50.9)	---
	During hospitalization	2 (12.7: 1.1–65.7)	2 (13.9: 1.2–68.3)	---
**Women diagnosed with HIV before or during the pregnancy and with a Pregnancy Booklet**		**N=9** **n (%: 95%CI)**	**N=9** **n (%: 95%CI)**	**N=0** **n (%: 95%CI)**
Viral load test performed		2 (26.6: 7.2–62.8)	2 (27.0: 7.3–63.4)	0
Receiving antiretroviral therapy		4 (40.0: 4.6–90.2)	4 (40.7%: 4.6–90.8)	0
**Delivery method in women with HIV hospitalized for childbirth**		**N=14** **n (%: 95%CI)**	**N=13** **n (%: 95%CI)**	**N=1** **n (%: 95%CI)**
Vaginal or forceps delivery		4 (31.2: 4.5–81.5)	4 (34.3: 4.5–85.1)	0
Cesarean section without labor		7 (45.2: 12.0–83.3)	6 (39.9: 8.3–83.0)	1 (100)
Cesarean section with labor		3 (23.5: 8.4–50.8)	3 (25.8: 9.8–52.7)	0

IC95%: 95% confidence interval;

* Due to data weighting, frequencies are generated with decimals and have been rounded to avoid being presented as case fractions. For this reason, the percentages shown in parentheses may be slightly different from those calculated using the numbers provided (numerator and denominator), especially for variables and categories with low frequencies.

Fifteen cases of HIV infection were identified, resulting in a prevalence of 0.79% (95%CI 0.31–1.99). Of these, 14 cases were among women with public funding (0.96; 95%CI 0.33–2.73) and one case was among women with private funding (0.27; 95%CI 0.05–1.50). The detection rate per 1,000 LB was 7.89 (95%CI 3.14–19.70) (Table 2). Most cases occurred in women who were already infected with HIV prior to the current pregnancy (79.7%), with 7.6% diagnosed during PN care and 12.7% diagnosed during hospital admission (Table 2).

**Figure 2:**
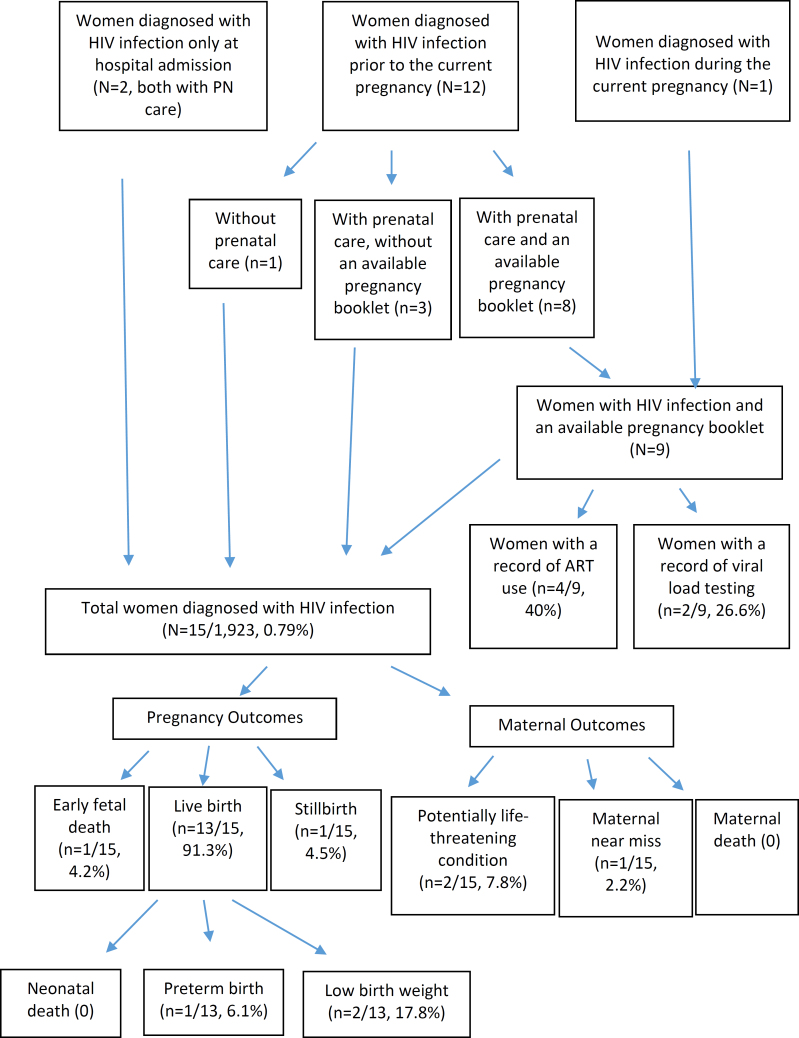
Indicators of management and outcomes of pregnancy and maternal health in women diagnosed with Human Immunodeficiency Virus (HIV). State of Rio de Janeiro, 2021–2023.

Of the nine women diagnosed with HIV infection prior to pregnancy or during PN care, and who had their PB data collected, 26.6% had a record of a viral load test during PN care, and 40% had a record of an ART prescription (Table 2, Figure 2).

Of the 14 women with HIV infection admitted for childbirth care, 45.2% underwent a cesarean section without labor, 23.5% had a cesarean section with labor, and 31.2% had a vaginal delivery (Table 2). Among the cesarean sections, 71.1% had a recorded indication for this mode of delivery due to HIV infection.

Of the 15 pregnancies diagnosed with HIV infection, 4.2% resulted in early fetal death and 4.2% in stillbirth. Among the LB, 6.1% were premature, and 17.8% had low birth weight. No neonatal deaths were reported. Regarding maternal outcomes, 7.8% experienced potentially life-threatening conditions, and 2.2% were classified as maternal near miss, with no cases of maternal death recorded (Table 3 and Figure 2). All negative outcomes were observed in women with public funding.

**Table 3:** Maternal and gestational outcomes in women with human immunodeficiency virus (HIV) infection, according to the type of hospital admission funding. State of Rio de Janeiro, 2021-2023!.

Indicators		Total (N=15) n (%: 95%CI)	Public funding (N=14) n (%: 95%CI)	Private funding (N=1) n (%: 95%CI)
**Maternal outcomes in women with HIV infection**				
	Potentially life-threatening condition*	2 (7.8: 0.9–43.7)	2 (8.5: 1.0–47.1)	0
	Maternal near miss*	1 (2.2: 0.1–33.8)	1 (2.4: 0.1–37.3)	0
	Maternal death*	0	0	0
**Pregnancy outcomes in women with HIV infection**				
	Early fetal loss	1 (4.2: 0.3–35.5)	1 (4.6: 0.4–37.9)	0
	Stillbirth	1 (4.5: 0.4–33.2)	1 (4.9: 0.5–35.2)	0
**Pregnancy outcomes in live births of women with HIV infection**		**N=13** **n (%: 95%CI)**	**N=12** **n (%: 95%CI)**	**N=1** **n (%: 95%CI)**
Neonatal death		0	0	0
Low birth weight		2 (17.8: 1.8–72.2)	2 (19.6: 2.0–74.6)	0
Prematurity		1 (6.1: 0.4–48.8)	1 (6.7: 0.5–52.1)	0

IC95%: 95% confidence interval;

* according to definitions by the World Health Organization; early fetal loss: fetal death with weight <500 g and gestational age <22 weeks; stillbirth: fetal death with weight ≥500 g or gestational age ≥22 weeks; neonatal death: death occurring up to the 27^th^ day of life; low birth weight: live birth with birth weight <2,500 g; prematurity: live birth with gestational age <37 weeks;

! due to data weighting, frequencies are generated with decimals and were rounded to avoid presenting them as fractions of cases. Therefore, the percentages presented in parentheses may be slightly different from those calculated based on the numbers presented (numerator and denominator), especially for variables and categories with low frequency.

## DISCUSSION

The results of this study revealed a prevalence of HIV infection in pregnant women of 0.79%, with no significant difference based on the type of hospital care financing. The majority of cases involved women diagnosed with HIV prior to the current pregnancy. Of the process indicators evaluated, only PN care coverage in postpartum women (in both public and private hospitals) and HIV testing during hospitalization for childbirth with public funding met the recommended target of 95%^1^. Women with public hospitalization had worse social conditions, less access to PN care services, later initiation of PN care, and fewer adequate consultations. Additionally, fewer second HIV tests were performed during PN care. Records from the PB showed low rates of viral load testing and ART use, while hospital data indicated a high proportion of cesarean sections.

The detection rate found in this study, 7.89 per 1,000 LB, is 1.5 times higher than the rate reported to the National System of Notifiable Diseases (*Sistema Nacional de Agravos de Notificação* – SINAN), which was 5.1 per 1,000 LB in 2021 and 2022 in RJ^7^, indicating an approximate underreporting of 35% of cases. Underreporting of HIV infection in pregnant women was also identified in a national study conducted between 2011 and 2012, where the search for information in other data systems led to a 57.1% increase in the number of cases reported to SINAN^14^. A potential explanation for the underreporting observed in this study, which could be explored in future research, is the failure to report women diagnosed with HIV prior to pregnancy, despite the recommendation that pregnant women with HIV be reported with each new pregnancy^7^. Another possibility is that the cases identified in this study were not subsequently confirmed, particularly if testing was conducted with only one rapid test, due to the potential for false-positive results.

No significant difference was observed in the prevalence of HIV infection based on the type of financing for hospital admission, although the point estimate for women with public financing was more than three times higher than that for women with private financing. It is possible that the small number of cases limited the study's power to detect this difference. Previous national studies with larger samples have shown a higher prevalence of HIV infection among women with worse social conditions^15^ and those treated in public services^16^.

The coverage of HIV testing in this study was lower than the estimated 88% observed in the Southeast Region between 2011 and 2012, the region where RJ^16^ is located. However, there was an increase in coverage of two HIV tests, rising from 33.3 to 46.1%. The *Nascer no Brasil I* study showed greater coverage of both the first and second HIV tests in private services between 2011 and 2012^16^, while in the present analysis, the highest testing coverage in the private sector was only observed for the second test. Lower HIV testing rates have been reported among pregnant women living in municipalities with poorer socioeconomic conditions^17^, in locations further away from PN care services^18–20^, among beneficiaries of the *Bolsa Família* Program^21^, among pregnant women of mixed race or black skin color^16,17,21^, and those with fewer years of education^16,17,21^. In contrast, higher testing coverage was observed among women who started PN care early, had an adequate number of PN appointments^16^, and received guidance on testing during PN care^22^. These findings highlight the importance of addressing women's social vulnerability and ensuring the availability of primary care services to improve access to HIV testing.

Testing during hospital admission was nearly universal in public services, but very low rates were observed in privately funded hospitalizations. One hypothesis for this result, which should be explored in future studies, is the care model in the private sector, where PN care and hospital care are generally provided by the same healthcare professional^23^. Current care guidelines recommend three HIV tests, with the third being conducted at hospital admission^13^. This underscores the importance of testing during hospitalizations for abortion care as a crucial opportunity to diagnose HIV infection in these women.

The higher proportion of women with HIV prior to pregnancy is consistent with national data showing that approximately 60% of women reported between 2021–2023 were diagnosed with HIV before the current pregnancy^7^. These results reflect the current context of reproductive health in women with HIV/AIDS, where improvements in clinical care and access to safe and effective medications to reduce VT have led to an increase in the number of pregnancies and LB in many countries^24–26^.

Viral load tests and the use of ART are integral components of care guidelines for managing HIV during pregnancy^13^. In this study, only a small proportion of women had records of these procedures in their PB. National data indicated that approximately 70% of pregnant women with HIV used ART during pregnancy in 2021/2022^7^, and our results were likely underestimated due to errors in PB documentation. Although national guidelines do not explicitly recommend recording viral load and ART results in the PB^13^, this information is essential for guiding childbirth care, with PB being the main link between PN care and hospital care.

In this study, nearly 70% of births occurred by cesarean section, with HIV infection recorded as the indication for this mode of delivery in most cases. At the national level, the proportion of cesarean sections was also higher than that of vaginal births, although the proportion of unknown birth types remains high among women with HIV in the country^7^. Local studies have similarly reported a high proportion of cesarean births in women with HIV^5,27^. Current recommendations suggest that elective cesarean sections should only be performed in pregnant women with an unknown viral load or a viral load greater than 1,000 copies from the 34^th^ week of gestation onward^13,28^. However, data from a cohort of women with HIV/AIDS in RJ indicate a high proportion of cesarean births, with no reduction observed between 2000 and 2016^29^, unlike in other countries where the proportion of vaginal births in women with HIV has increased^30,31^. In a country with a high rate of cesarean sections, these results may reflect both a lack of knowledge regarding healthcare guidelines, leading to an increase in indications for cesarean sections in women with HIV, and the absence of viral load tests close to delivery. In a national study conducted between 2011 and 2012^4^, viral load data were unavailable for 45% of pregnant women.

The small number of women with HIV limited the analysis of gestational and maternal outcomes, with all unfavorable outcomes occurring in publicly funded hospitalizations. The proportion of early fetal losses and stillbirths was similar to the national data recorded between 2021 and 2023^7^. The point estimate for the proportion of low birth weight (17.8%) was high, exceeding the national average of 9.4% for the total number of LB in 2022, while the point estimate for preterm births (6.1%) was lower than the national average (11.8%). However, the wide confidence interval does not allow for definitive conclusions about the statistical significance of these differences. A higher prevalence of unfavorable perinatal outcomes has been reported in pregnant women with HIV, regardless of income level or region of residence^32^. The proportion of potentially life-threatening conditions and maternal near misses found in this study aligns with estimates for Brazil^33^, with no cases of maternal death identified, a rarer outcome. Previous studies have reported an increased risk of severe maternal morbidity associated with HIV infection^34–36^.

This study has some limitations. Women admitted to hospitals with fewer than 100 births/year or those with non-hospital births were not included, meaning the results are not applicable to this population. In the assessment of PN care, the absence of test and treatment records in the PB was considered a lack of performance. While this approach may have overestimated the proportion of untested and untreated women, it is understood that the PB should be thoroughly completed. Finally, data on the use of injectable zidovudine, prophylaxis for exposed children, inhibition of lactation, and provision of milk formula, as well as information on newborn blood tests, were unavailable. These gaps limited the evaluation of hospital management for both women and their babies and prevented the calculation of VT rates.

In conclusion, missed opportunities were identified in the management of pregnant women with HIV in both public and private hospitals in RJ. The detection rate found in this study was higher than that recorded in SINAN, suggesting issues with case reporting. Women with public funding for hospital care demonstrated greater social vulnerability and had less access to prenatal care and testing during pregnancy, with all adverse gestational and maternal outcomes occurring among this group.

## Supplementary Material


