# Supplementary material for: HIV infection during pregnancy in the state of Rio de Janeiro, Brazil, 2021-2023
Source: Rev Bras Epidemiol. 2025 May 9;28:e250020. doi: 10.1590/1980-549720250020 (PMC12068811; doi:10.1590/1980-549720250020)
Supplement: Supplementary file 1 [file 1980-5497-rbepid-28-e250020-supll1.pdf]

Tabela S1 - Material Suplementar

Universo de hospitais e de Nascidos Vivos (NV) e amostra de hospitais, NV e puérperas de parto no estudo Nascer no Brasil II (NBII) no estado do Rio de Janeiro.

			Universo		Amostra no estudo NBII		
**Tipo**	**Tamanho**	**Local**	**Hospitais***	**NV****	**Hospitais**	**NV**	**Puérperas**
Público (13)	≥500 (10)	RM	33	108,153	7	619	622
		Interior	13	19,372	3	266	268
	100-499 (3)	RM	5	1,548	2	59	59
		Interior	12	2,616	1	30	30
Misto (3)	≥500 (2)	RM	3	4,596	1	49	49
		Interior	15	23,673	1	89	90
	100-499 (1)	RM	0	0	0	0	0
		Interior	13	2,800	1	30	30
Privado (13)	≥500 (12)	RM	25	47,108	10	480	483
		Interior	6	4,478	2	100	101
	100-499 (1)	RM	7	2,269	1	30	30
		Interior	14	4,486	0	0	0
**Total**			**146**	**221,099**	**29**	**1,752**	**1,762**

* Universo de hospitais elegíveis, ou seja, com pelo menos 100 NV em 2022, segundo SINASC-2022

** Número de NV em 2022 nos hospitais elegíveis do estrato, segundo SINASC-2022

Nota: Não houve cálculo de tamanho amostral para mulheres internadas com diagnóstico de abortamento, sendo elegíveis todas aquelas admitidas durante o período de tempo necessário para o alcance da amostra planejada de puérperas de parto em cada unidade hospitalar.

A ampliação da amostra foi realizada nos hospitais públicos devido ao maior volume de partos nessas unidades, visando equilibrar a proporção amostral relativa. Nos hospitais mistos, a expansão se justificou pelo pequeno número de unidades selecionadas, aumentando o poder da amostra. Já nos hospitais privados, a ampliação da amostra não foi necessária, pois a proporção de puérperas amostradas em relação ao universo já era suficientemente elevada.
