# Supplementary material for: HIV infection during pregnancy in the state of Rio de Janeiro, Brazil, 2021-2023
Source: Rev Bras Epidemiol. 2025 May 9;28:e250020. doi: 10.1590/1980-549720250020 (PMC12068811; doi:10.1590/1980-549720250020)
Supplement: Translation

# Translation: Infecção pelo HIV na gestação no estado do Rio de Janeiro, Brasil, 2021–2023

**DOI:** 10.1590/1980-549720250020.2

**Authors:** Rosa Maria Soares Madeira Domingues, Marcos Augusto Bastos Dias, Ana Paula Esteves-Pereira, Paula Mendes Luz, Emilia Jalil, Vania Rocha, Angela Cristina Vasconcelos de Andrade Rabello, Ruth Khalili Friedman, Maria do Carmo Leal

**Affiliations:** I Fundação Oswaldo Cruz, Instituto Nacional de Infectologia Evandro Chagas – Rio de Janeiro (RJ), Brasil.; II Fundação Oswaldo Cruz, Instituto Nacional da Mulher, da Criança e do Adolescente Fernandes Figueira – Rio de Janeiro (RJ), Brasil.; III Fundação Oswaldo Cruz, Escola Nacional de Saúde Pública Sérgio Arouca – Rio de Janeiro (RJ), Brasil.

**Keywords:** Gravidez, Infecções por HIV, Cuidado Pré-Natal, Avaliação em Saúde

## Abstract

**Objetivos:**

Estimar a prevalência de infecção pelo vírus da imunodeficiência humana (HIV) em gestantes; analisar os desfechos gestacionais e maternos das mulheres com HIV; e avaliar indicadores de processo para prevenção da transmissão vertical do HIV segundo tipo de financiamento da internação hospitalar no estado do Rio de Janeiro.

**Métodos:**

Estudo seccional, com 1.923 mulheres, realizado no período 2021–2023. Foram realizadas entrevistas com mulheres e extraídos dados da caderneta da gestante e de prontuários hospitalares. Foi estimada a prevalência de infecção pelo HIV, dos desfechos gestacionais e maternos e da adequação de indicadores de processo do manejo do HIV com respectivos intervalos de confiança de 95% (IC95%) segundo tipo de financiamento (público ou privado) da internação hospitalar para parto ou abortamento.

**Resultados:**

Cobertura de assistência pré-natal, de realização de um e dois testes de HIV no pré-natal e de testagem na internação hospitalar de 93,7, 79,7, 45,8 e 78,2%, respectivamente. A prevalência de infecção pelo HIV foi estimada em 0,79% (IC95% 0,31–1,99). Apenas 40% das mulheres com HIV apresentavam registro de tratamento antirretroviral e 26% registro de exame de carga viral na caderneta da gestante. Mulheres com internação hospitalar com financiamento público eram mais vulneráveis socialmente e apresentaram menor cobertura de assistência pré-natal e de testagem com dois testes anti-HIV.

**Conclusão:**

Foram identificadas oportunidades perdidas no manejo das gestantes com HIV em internações com financiamento público e privado no Rio de Janeiro. A taxa de detecção foi superior à do Sistema de Informação de Agravos de Notificação e sugere subnotificação de casos.

## INTRODUÇÃO

A transmissão vertical (TV) do vírus da imunodeficiência humana (HIV), de mãe para filho durante a gestação, parto ou aleitamento materno, é a principal forma de infecção de crianças e pode ser evitada com os recursos assistenciais disponíveis^1^, entretanto falhas no acesso aos serviços e na integração destes ameaçam o alcance das metas globais de eliminação da TV do HIV^2,3^. No Brasil, recursos e diretrizes assistenciais para manejo do HIV na gestação estão disponíveis desde os anos 1990, mas ainda existem problemas na implementação dessas medidas, com oportunidades perdidas de prevenção da TV^4–6^.

A taxa de detecção de HIV em gestantes no Brasil apresentou valor de 3,1 por 1.000 nascidos vivos (NV) em 2022, enquanto a taxa de detecção de aids em crianças menores de 5 anos de idade, utilizada para monitoramento da TV, foi de 1,5 caso/100 mil habitantes. Nesse mesmo ano, o estado do Rio de Janeiro foi a unidade da federação com a terceira maior taxa nacional de detecção de HIV em gestantes (5,1 por 1.000 NV) e de detecção de aids em menores de 5 anos (3,8 por 100.000 habitantes)^7^.

Estudos prévios realizados no município do Rio de Janeiro e na região metropolitana do estado evidenciaram falhas na testagem de gestantes durante a assistência pré-natal (PN)^8^ e na cascata de cuidados PN, perinatal e pós-natal^9^.

Este estudo tem como objetivos:

- estimar a prevalência de infecção pelo HIV em gestantes;
- analisar os desfechos gestacionais e maternos das mulheres com infecção pelo HIV e das crianças expostas ao HIV;
- avaliar alguns indicadores de processo para prevenção da TV, segundo tipo de financiamento da internação hospitalar para parto ou aborto no RJ, utilizando dados da pesquisa Nascer no Brasil II.

## Métodos

Este estudo é parte da pesquisa "Nascer no Brasil II: pesquisa nacional sobre aborto, parto e nascimento (NBII)".

### A pesquisa NBII

Estudo transversal, de base hospitalar e âmbito nacional. Foi selecionada uma amostra probabilística em dois estágios (hospitais e mulheres e seus recém-nascidos), sendo os hospitais estratificados por macrorregião, localização (região metropolitana ou interior), tipo (público, misto, privado) e tamanho (100-499, ≥500 NV/ano). Foram entrevistadas 30 e 50 puérperas por hospitais selecionados com 100–499 e ≥500 NV/ano, respectivamente.

Foram elegíveis puérperas de NV com qualquer peso ou idade gestacional (IG), puérperas de natimortos com IG≥22 semanas ou peso ≥500 g, e mulheres internadas com diagnóstico de abortamento. Não foram elegíveis mulheres com parto não hospitalar; com parto trigemelar ou mais; com dificuldade de comunicação; que receberam alta ainda gestante; e as internadas para realização de parto por determinação judicial.

Foram realizadas entrevistas no puerpério imediato e extraídos dados das cadernetas da gestante e do prontuário hospitalar. O protocolo do estudo NBII se encontra publicado em Leal et al.^10^.

### Estudo do estado do Rio de Janeiro

A amostra de puérperas do estado do RJ foi calculada utilizando-se os mesmos parâmetros do estudo nacional: proporção de cesarianas (57% no RJ em 2019), nível de significância de 5% e poder de 90% para detectar diferenças de 7%. Utilizou-se um efeito de desenho de 1,3, resultando no planejamento de uma amostra mínima de 1.350 mulheres. Para alcançar esse quantitativo, houve ampliação da amostra, de 50 para 90 puérperas nos hospitais públicos e mistos com ≥500 NV/ano (Mat. Supl. 1: https://doi.org/10.7303/syn64828293.1). As entrevistas hospitalares ocorreram entre novembro de 2021 e junho de 2023 em 29 hospitais de 18 municípios (13 públicos em 12 municípios, três mistos em três municípios e 13 privados em seis municípios).

### Variáveis e fonte de dados

Foram analisados os seguintes indicadores baseados em recomendações da Organização Mundial da Saúde (OMS)^1,11,12^ e do Ministério da Saúde brasileiro^13^:

- Assistência PN: ter recebido ao menos uma consulta de PN^1^;
- Recebimento da caderneta da gestante (CG) durante a assistência PN^11^;
- Início precoce: primeira consulta PN até a 12ª semana gestacional^11^;
- Adequação do número de consultas de PN: número de consultas para a IG no parto considerando o calendário e o número mínimo de oito consultas recomendado pela OMS^11^;
- Realização de testagem para o HIV na gestação: primeira testagem no início do PN e segunda testagem da 28ª semana de gestação em diante^13^, analisada apenas em mulheres com IG≥34 semanas no momento do parto;
- Testagem para HIV na internação hospitalar para assistência ao parto ou abortamento^13^;
- Prevalência de infecção pelo HIV na gestação e taxa de detecção por 1.000 NV: infecção pelo HIV definida pelo registro de diagnóstico anterior à gestação atual e/ou testagem na assistência PN e/ou na internação hospitalar com resultado reagente e/ou diagnóstico de infecção pelo HIV durante a gestação, incluindo registros de diagnóstico durante a gestação, causa da internação, indicação de cesariana ou diagnóstico de criança exposta ao HIV. Casos foram classificados segundo o momento do diagnóstico (anterior à assistência PN, na assistência PN, na admissão hospitalar). A prevalência de infecção pelo HIV foi calculada considerando-se todas as gestações no denominador, enquanto a taxa de detecção foi calculada em relação ao número de NV^7^;
- Realização de exame de carga viral para o HIV durante a assistência PN: proporção de gestantes com HIV com registro de resultado de exame de carga viral durante a assistência PN^13^;
- Tratamento com terapia antirretroviral (TARV) durante a gestação: proporção de gestantes com HIV com registro de prescrição de TARV durante a gestação^11,13^;
- Via de nascimento: proporção de parturientes com HIV que apresentaram parto vaginal, cesariana sem trabalho de parto e cesariana com trabalho de parto;
- Desfecho materno: proporção de gestantes com HIV classificadas como caso de condição potencialmente ameaçadora à vida, *near miss* materno e óbito materno, segundo definições da OMS^12^;
- Desfecho da gestação: proporção dos desfechos "perda fetal precoce" (óbito fetal com peso<500 g e IG<22 semanas), "natimorto" (óbito fetal com peso≥500 g ou IG≥22 semanas), "óbito neonatal" (óbito ocorrido até o 27° dia de vida), prematuridade (NV com menos de 37 semanas gestacionais) e baixo peso ao nascer (NV com peso ao nascer <2.500 g) em mulheres com HIV na gestação.

Como características maternas, foram analisadas as variáveis: "tipo de financiamento da internação" ("público": mulheres atendidas em hospital público ou hospital privado com internação paga pelo Sistema Único de Saúde; "privado": mulheres atendidas em hospital privado com pagamento da internação por plano de saúde ou desembolso direto); idade (<20, 20 a 34, 35 anos ou mais); raça/cor da pele (branca, parda e preta); anos completos de estudo (≤8, 9 a 11, 12 a 15 e 16 anos ou mais); e situação conjugal (vive ou não com companheiro).

Dados relativos às características maternas e ao recebimento da CG, época de início do PN e número de consultas foram obtidos na entrevista hospitalar com a mulher. Dados relativos a exames e prescrição de TARV realizados no PN foram extraídos da CG, enquanto exames na internação hospitalar e desfechos da gestação foram obtidos do prontuário hospitalar. Exames e medicamentos não registrados na CG nem no prontuário hospitalar foram considerados não realizados.

### Análise dos dados

Todas as análises foram realizadas para a amostra total e categorizadas segundo tipo de financiamento da internação hospitalar. Para descrição das características das mulheres e estimativa de prevalência de infecção pelo HIV, testagem para HIV na internação hospitalar e desfechos maternos e gestacionais, foram incluídas todas as mulheres. Para a análise dos indicadores relativos a exames e medicamentos utilizados durante a assistência PN, foram excluídas as mulheres que não tiveram a CG digitada.

Inicialmente, foram analisadas as características demográficas e sociais das mulheres e posteriormente estimados os indicadores de manejo da infecção pelo HIV na gestação com os respectivos intervalos de confiança de 95% (IC95%). Foi adotado o padrão de referência≥95%, recomendado pela OMS para indicadores de processo para prevenção da TV do HIV^1^.

As análises foram realizadas com o *software* IBM Statistical Package for the Social Sciences Statistics for Windows, Versão 19.0 (IBM Corp., Armonk, NY, Estados Unidos da América), com inclusão do efeito de desenho, ponderação e calibração da amostra.

A pesquisa NBII foi aprovada pela Comissão Nacional de Ética em Pesquisa, Certificado de Apresentação de Apreciação Ética: 21633519.5.0000.5240, em 22 de junho de 2020, e teve aprovação dos comitês locais de ética em pesquisa das instituições ou da diretoria clínica quando da ausência dos comitês locais.

## RESULTADOS

Menos de 5% das mulheres eram não elegíveis, recusaram a participação na pesquisa ou foram perdidas por alta hospitalar antes da entrevista, sendo repostas no mesmo hospital. Foram entrevistadas 1.923 mulheres, sendo 91,6% (1.762/1.923) por internação para o parto e 8,4% (161/1.923) por diagnóstico de abortamento. Aproximadamente um quarto das mulheres teve financiamento privado da assistência hospitalar (477/1.923). A maior parte das mulheres tinha de 20 a 34 anos, se declarou como parda, apresentava 12 ou mais anos de estudo e vivia com companheiro. A cobertura de PN foi de 93,7% (1.802/1.923), com proporção significativamente mais baixa naquelas em internação pós-aborto (41,8%, 67/161). Em mulheres com PN, 98,1% (1.767/1.802) receberam CG, 77,8% (1.401/1.802) tiveram início precoce do acompanhamento PN e 71,5% (1.289/1.802) o número adequado de consultas. Em mulheres com financiamento público, observou-se maior proporção de adolescentes (idade<20 anos), pardas e pretas, e com até 11 anos de estudo, enquanto entre aquelas com financiamento privado houve maior proporção de mulheres com 35 anos ou mais, brancas, com 16 ou mais anos de estudo e que viviam com companheiro. Mulheres com financiamento privado apresentaram maior cobertura de PN, maior proporção de início precoce e adequação do número de consultas e menor proporção de recebimento da CG (Tabela 1).

**Tabela 1:** Características sociais, demográficas e de utilização do pré-natal segundo tipo de financiamento da internação hospitalar. Estado do Rio de Janeiro, 2021–2023.

Característica		Total (N=1.923) n (%: IC95%)	Financiamento público (N=1.446) n (%: IC95%)	Financiamento privado (N=477) n (%: IC95%)
**Tipo de puérpera**				
	Pós-Parto	1762 (91,6: 88,9–93,7)	1.305 (90,2: 87,8–92,2)	457 (95,8: 86,3-–98,8)
	Aborto	161 (8,4: 6,3–11,1)	141 (9,8: 7,8–12,2)	20 (4,2: 1,2–13,7)
**Idade materna (anos)**				
	<20	188 (9,7: 7,8–12,2)	181 (12,5: 10,7–14,5)	7 (1,4: 0,5–3,8)
	20 a 34	1.362 (70,8: 68,5–73,1)	1.055 (73,0: 69,8–75,9)	307 (64,4: 59,3–69,1)
	35 e mais	373 (19,4: 17,2–21,8)	210 (14,5: 12,9–16,4)	163 (34,2: 29,1–39,7)
**Cor da pele**				
	Branca	569 (29,6: 25,0–34,6)	331 (22,9: 18,6–27,8)	238 (50,0: 43,8–56,2)
	Preta	390 (20,3: 16,7–24,4)	346 (23,9: 20,8–27,4)	44 (9,2: 6,5–12,9)
	Parda	964 (50,1: 46,7–53,5)	769 (53,2: 48,7–57,6)	195 (40,8: 34,0–48,0)
**Anos de estudo**				
	≤8	290 (15,1: 12,5–18,1)	286 (19,8: 17,2–22,6)	4 (0,9: 0,5–1,6)
	9 a 11	538 (28,0: 21,2–35,9)	499 (34,5: 28,5–41,1)	39 (8,1: 4,8–13,4)
	12 a 15	789 (41,0: 35,5–46,8)	586 (40,5: 33,7–47,7)	203 (42,5: 34,6–50,9)
	16 ou mais	306 (15,9: 11,3–21,8)	75 (5,2: 3,6–7,2)	231 (48,4: 37,3–59,6)
**Vive com o companheiro**		1.535 (79,9: 75,6–83,6)	1.089 (75,4: 71,8–78,7)	446 (93,5: 89,6–96,0)
**Fez pré-natal (uma ou mais consultas)**		1.802 (93,7: 91,8–95,2)	1.332 (92,1: 90,0–93,8)	470 (98,5: 95,9–99,4)
	Mulheres pós-parto (total=1.762, público=1.305, privado=457)	1.735 (98,5: 97,5–99,1)	1.278 (98,0: 96,5–98,8)	456 (99,9: 98,9–100)
	Mulheres pós-aborto (total=161, público=141, privado=20)	67 (41,8: 32,7–51,4)	54 (38,3: 30,2–47,2)	13 (66,3: 53,8–76,9)
**Mulheres com assistência pré-natal**		**(N=1.802)** **n (%: IC95%)**	**(N=1.332)** **n (%: IC95%)**	**(N=470)** **n (%: IC95%)**
**Recebeu caderneta da gestante**		1.767 (98,1: 96,3–99,0)	1.325 (99,5: 98,1–99,8)	442 (94,2: 90,0–96,6)
**Início precoce (até 12 semanas)**		1.401 (77,8: 74,7–80,5)	963 (72,3: 69,0–75,3)	438 (93,3: 90,8–95,2)
**Número adequado de consultas**		1.289 (71,5: 68,4–74,5)	898 (67,4: 63,3–71,3)	391 (83,2: 76,7–88,1)

IC95%: Intervalo de confiança de 95%.

Do total de entrevistadas, 70,3% (1.353/1.923) teve dados da CG digitados (Figura 1), com menor proporção entre mulheres com financiamento privado (60,9% [255/418] privado *vs*. 84,8% [1.098/1.295] público, p<0,001). Das mulheres com CG digitadas, 79,7 (1.078/1.353) e 45,8% (595/1.298) tiveram a primeira e a segunda testagem para HIV realizadas durante o PN, respectivamente. Na internação hospitalar, a testagem para HIV foi registrada em 78,2% (1.504/1.923) dos prontuários, sem diferença significativa em internações para assistência ao parto ou ao abortamento. Maior cobertura da segunda testagem para HIV durante o PN foi observada em mulheres com financiamento privado, enquanto maior cobertura de testagem na internação hospitalar foi observada em mulheres com financiamento público, tanto em internações para parto (97,7% [1.274/1.305] público, 20,7% [95/457 privado) quanto por aborto (93,3% [132/141] público, 17,7% [3/20] privado) (Tabela 2).

**Figura 1:** Fluxograma de mulheres analisadas no estudo e estimativa de gestantes com diagnóstico de infecção pelo vírus da imunodeficiência humana (HIV). Estado do Rio de Janeiro, 2021–2023.

**Tabela 2:** Prevalência de infecção pelo vírus da imunodeficiência humana (HIV) na gestação e indicadores de manejo da infecção pelo HIV, segundo tipo de financiamento da internação hospitalar. Estado do Rio de Janeiro, 2021–2023*.

Indicadores		Total	Financiamento público	Financiamento privado
**Mulheres com caderneta da gestante**		**N=1.353** **n (%: IC95%)**	**N=1.098** **n (%: IC95%)**	**N=255** **n (%: IC95%)**
Primeira sorologia para HIV no pré-natal		1078 (79,7: 73,5–84,7)	887 (80,7%: 73,5–86,3)	191 (75,1: 69,0–80,4)
**Mulheres com caderneta da gestante e idade gestacional no parto ≥34 semanas**		N=1.298 n (%: IC95%)	N=1.047 n (%: IC95%)	N=251 n (%: IC95%)
Segunda sorologia para HIV no pré-natal		595 (45,8: 39,0–52,7)	452 (43,2: 34,9–51,9)	143 (56,8: 47,1–66,0)
**Total de mulheres**		**(N=1.923)** **n (%: IC95%)**	**(N=1.446)** **n (%: IC95%)**	**(N=477)** **n (%: IC95%)**
**Sorologia para HIV na internação hospitalar (pós-parto ou abortamento)**		1.504 (78,2: 67,2–86,3)	1.406 (97,2: 93,0–98,9)	98 (20,6: 14,2–28,8)
	Pós-parto (total=1.762, público=1.305, privado=457)	1.369 (77,7: 66,7–85,8)	1.274 (97,7: 93,1–99,2)	95 (20,7: 14,1–29,3)
	Abortamento (total=161, público=141, privado=20)	135 (83,7: 65,1–93,4)	132 (93,0: 80,3–97,8)	3 (17,7: 5,9–42,8)
**Prevalência de infecção pelo HIV**		15 (0,79: 0,31–1,99)	14 (0,96: 0,33–2,73)	1 (0,27: 0,05–1,50)
	Mulheres com admissão para parto (total=1.762, público=1.305, privado=457)	14 (0,82: 0,32–2,08)	13 (1,01: 0,35–2,90)	1 (0,28: 0,05–1,54)
	Mulheres com admissão por abortamento (total=161, público=141, privado=20)	1 (0,39: 0,05–3,13)	1 (0,45: 0,05–3,60)	0
	Taxa de detecção por 1.000 nascidos vivos (total=1.752, público=1.295, privado=457)	15 (7,89: 3,14–19,70)	14 (9,68: 3,38–27,42)	1 (2,81:0,51–15,4)
**Mulheres com diagnóstico de HIV**		**N=15** **n (%: IC95%)**	**N=14** **n (%: IC95%)**	**N=1** **n (%: IC95%)**
**Momento do diagnóstico da infecção**				
	Antes da gestação atual	12 (79,7: 18,9–98,5)	11 (77,8: 16,6–98,4)	1 (100: 100–100)
	Durante a assistência pré-natal	1 (7,6:0,7–48,4)	1 (8,3: 0,8–50,9)	---
	Na internação hospitalar	2 (12,7: 1,1–65,7)	2 (13,9: 1,2–68,3)	---
**Mulheres com diagnóstico de HIV antes ou ao longo da gestação e com caderneta da gestante**		**N=9** **n (%: IC95%)**	**N=9** **n (%: IC95%)**	**N=0** **n (%: IC95%)**
Realização de carga viral		2 (26,6: 7,2–62,8)	2 (27,0: 7,3–63,4)	0
Recebimento de tratamento com terapia antirretroviral		4 (40,0: 4,6–90,2)	4 (40,7%: 4,6–90,8)	0
**Via de nascimento em mulheres com infecção pelo HIV internadas para parto**		**N=14** **n (%: IC95%)**	**N=13** **n (%: IC95%)**	**N=1** **n (%: IC95%)**
Parto vaginal ou fórceps		4 (31,2: 4,5–81,5)	4 (34,3: 4,5–85,1)	0
Cesariana sem trabalho de parto		7 (45,2: 12,0–83,3)	6 (39,9: 8,3–83,0)	1 (100)
Cesariana com trabalho de parto		3 (23,5: 8,4–50,8)	3 (25,8: 9,8–52,7)	0

IC95%: intervalo de confiança 95%;

* por causa da ponderação de dados, as frequências são geradas com decimais e foram arredondadas para não serem apresentadas como fração de casos. Por essa razão, os percentuais apresentados entre parênteses podem ser um pouco diferentes dos calculados por meio dos números apresentados (numerador e denominador), principalmente para variáveis e categorias com frequência baixa.

Foram identificados 15 casos de infecção pelo HIV com prevalência de 0,79% (IC95% 0,31–1,99), sendo 14 casos em mulheres com financiamento público (0,96; IC95% 0,33–2,73) e um caso entre aquelas com financiamento privado (0,27; IC95% 0,05–1,50). A taxa de detecção por 1.000N V foi de 7,89 (IC95% 3,14–19,70) (Tabela 2). A maior parte dos casos era de mulher com infecção pelo HIV anterior à gestação atual (79,7%), sendo 7,6% diagnosticados durante a assistência PN e 12,7% durante a internação hospitalar (Tabela 2).

Das nove mulheres com diagnóstico da infecção pelo HIV anterior à gestação ou durante o PN e que tinham CG digitada, 26,6% apresentavam registro de exame de carga viral durante o PN e 40% o registro de prescrição de TARV (Tabela 2, Figura 2).

**Figura 2:** Indicadores de manejo e desfechos da gestação e maternos de mulheres com diagnóstico de infecção pelo vírus da imunodeficiência humana (HIV). Estado do Rio de Janeiro, 2021–2023.

Das 14 mulheres com infecção pelo HIV admitidas para assistência ao parto, 45,2% tiveram cesariana sem trabalho de parto, 23,5% cesariana com trabalho de parto e 31,2% parto vaginal (Tabela 2). Em 71,1% das cesarianas, havia registro de indicação dessa via de nascimento por causa da infecção pelo HIV.

Das 15 gestações com diagnóstico de infecção pelo HIV, 4,2% resultaram em óbito fetal precoce e 4,2% em natimorto. Dos NV, 6,1% foram prematuros e 17,8% apresentaram baixo peso ao nascer. Não foram identificados óbitos neonatais. Em relação aos desfechos maternos, 7,8% apresentaram condição potencialmente ameaçadora à vida e 2,2% foram caso de *near miss* materno, não tendo sido registrado caso de óbito materno (Tabela 3 e Figura 2). Todos os desfechos negativos foram observados em mulheres com financiamento público.

**Tabela 3:** Desfechos maternos e da gestação em mulheres com infecção pelo vírus da imunodeficiência humana (HIV), segundo tipo de financiamento da internação hospitalar. Estado do Rio de Janeiro, 2021-2023!.

Indicadores		Total (N=15) n (%: IC95%)	Financiamento público (N=14) n (%: IC95%)	Financiamento privado (N=1) n (%: IC95%)
**Desfechos maternos em mulheres com infecção pelo HIV**				
	Condição potencialmente ameaçadora à vida*	2 (7,8: 0,9–43,7)	2 (8,5: 1,0–47,1)	0
	*Near miss* materno*	1 (2,2: 0,1–33,8)	1 (2,4: 0,1–37,3)	0
	Óbito materno*	0	0	0
**Desfechos da gestação em mulheres com infecção pelo HIV**				
	Perda fetal precoce	1 (4,2: 0,3–35,5)	1 (4,6: 0,4–37,9)	0
	Natimorto	1 (4,5: 0,4–33,2)	1 (4,9: 0,5–35,2)	0
**Desfechos da gestação em nascidos vivos de mulheres com infecção pelo HIV**		**N=13** **n (%: IC95%)**	**N=12** **n (%: IC95%)**	**N=1** **n (%: IC95%)**
Óbito neonatal		0	0	0
Baixo peso ao nascer		2 (17,8: 1,8–72,2)	2 (19,6: 2,0–74,6)	0
Prematuridade		1 (6,1: 0,4–48,8)	1 (6,7: 0,5–52,1)	0

IC95%: intervalo de confiança de 95%;

* segundo definições da Organização Mundial da Saúde; perda fetal precoce: óbito fetal com peso<500 g e idade gestacional<22 semanas; natimorto: óbito fetal com peso≥500 g ou idade gestacional≥22 semanas; óbito neonatal: óbito ocorrido até o 27° dia de vida; baixo peso ao nascer: nascido vivo com peso ao nascer<2.500 g; prematuridade: nascido vivo com idade gestacional<37 semanas;

! por causa da ponderação de dados, as frequências são geradas com decimais e foram arredondadas para não serem apresentadas como fração de casos. Por essa razão, os percentuais apresentados entre parênteses podem ser um pouco diferentes dos calculados por meio dos números apresentados (numerador e denominador), principalmente para variáveis e categorias com frequência baixa.

## DISCUSSÃO

Os resultados deste estudo evidenciaram prevalência de infecção pelo HIV em gestantes de 0,79%, sem diferença significativa segundo tipo de financiamento da assistência hospitalar, sendo a maior parte dos casos de mulheres com diagnóstico anterior à gestação atual. Dos indicadores de processo avaliados, apenas a cobertura de PN em mulheres pós-parto (em internações públicas e privadas) e a testagem na internação para parto com financiamento público alcançaram a meta recomendada de 95%^1^. Mulheres com internação hospitalar com financiamento público apresentaram piores condições sociais, menor acesso a serviços de PN, com início mais tardio e menor adequação do número de consultas, além de menor realização da segunda testagem para o HIV durante o PN. Os registros na CG mostraram baixa realização de exames de carga viral e de uso de TARV, enquanto dados hospitalares revelaram elevada proporção de cesarianas.

A taxa de detecção encontrada neste estudo, de 7,89 por 1.000 NV, é 1,5 vez superior à reportada ao Sistema Nacional de Agravos de Notificação (SINAN), de 5,1 por 1.000 NV em 2021 e 2022 no RJ^7^, correspondendo a uma subnotificação aproximada de 35% dos casos. Subnotificação da infecção pelo HIV em gestantes foi evidenciada em estudo nacional realizado entre 2011 e 2012, em que a busca de informações em outros sistemas de informação resultou em aumento de 57,1% do número de casos notificados no SINAN^14^. Uma possível hipótese para a subnotificação encontrada neste estudo, a ser investigada em estudos futuros, é a não notificação de mulheres com diagnóstico anterior à gestação, apesar da recomendação de que gestantes com HIV sejam notificadas a cada nova gestação^7^. Outra hipótese consiste na não confirmação posterior dos casos identificados neste estudo, principalmente se a testagem foi realizada com apenas um teste rápido, por causa da possibilidade de resultados falso-positivos.

Não foi observada diferença significativa na prevalência de infecção pelo HIV segundo tipo de financiamento da internação hospitalar, embora a estimativa pontual em mulheres com financiamento público tenha sido mais de três vezes superior à observada entre aquelas com financiamento privado. É possível que o pequeno número de casos tenha limitado o poder do estudo para detectar essa diferença, ressaltando-se que em estudos nacionais anteriores, com amostras maiores, a prevalência de infecção pelo HIV foi mais elevada em mulheres com pior condição social^15^ e naquelas atendidas em serviços públicos^16^.

A cobertura de realização de um teste para o HIV foi inferior à estimativa de 88% observada na Região Sudeste entre 2011 e 2012, região do país onde está localizado o RJ^16^, entretanto houve aumento de cobertura de dois testes, passando de 33,3 para 46,1%. O estudo Nascer no Brasil I evidenciou maior cobertura da primeira e da segunda testagem para o HIV em serviços privados entre 2011 e 2012^16^, enquanto na presente análise a maior cobertura de testagem no setor privado foi verificada apenas para o segundo teste. Menor testagem para HIV já foi relatada em gestantes residentes em municípios com pior condição socioeconômica^17^, em locais mais distantes dos serviços de PN^18–20^, em beneficiárias do Programa Bolsa Família^21^, em gestantes de cor parda ou preta^16,17,21^ e com menos anos de estudo^16,17,21^, enquanto maior cobertura foi observada em mulheres com início precoce e número adequado de consultas de PN^16^ e naquelas com orientações recebidas sobre a testagem durante o PN^22^. Esses dados reforçam a importância da vulnerabilidade social das mulheres e da disponibilidade de serviços de atenção primária para o acesso à testagem para o HIV.

A testagem na internação hospitalar foi praticamente universal em serviços públicos, porém valores muito baixos foram observados em internações com financiamento privado. Uma hipótese para esse resultado, a ser investigada em estudos futuros, é o modelo de atenção nesse setor, cuja assistência PN e hospitalar é geralmente realizada pelo mesmo profissional de saúde^23^. As diretrizes assistenciais vigentes recomendam três testagens para o HIV, sendo a terceira na admissão hospitalar^13^, destacando-se a relevância da testagem em internações para assistência ao abortamento como uma oportunidade de diagnóstico da infecção pelo HIV nessas mulheres.

A maior proporção de mulheres com HIV anterior à gestação é coerente com dados nacionais que mostram que aproximadamente 60% das mulheres notificadas no período 2021–2023 tiveram diagnóstico da infecção antes da gestação atual^7^. Esses resultados refletem o contexto atual de saúde reprodutiva em mulheres com HIV/aids, em que o melhor cuidado clínico e acesso a medicamentos seguros e eficazes para a redução da TV resultou em aumento do número de gestações e de NV em muitos países^24–26^.

A realização de exames de carga viral e a utilização de TARV fazem parte das diretrizes assistenciais para manejo do HIV na gestação^13^. Neste estudo, uma proporção muito pequena de mulheres apresentava registro desses procedimentos na CG. Dados nacionais mostraram utilização de TARV na gestação por aproximadamente 70% das gestantes com HIV em 2021/2022^7^, estando nosso resultado provavelmente subestimado por falhas no registro da CG. Embora não exista uma recomendação explícita das diretrizes nacionais para registro de resultados de carga viral e TARV na CG^13^, ressalta-se a importância dessas informações para a definição de condutas na assistência ao parto, sendo a CG o principal elo entre a assistência PN e hospitalar.

Neste estudo, quase 70% dos nascimentos ocorreram por cesariana, com a infecção pelo HIV registrada como indicação dessa via de nascimento na maioria dos casos. No âmbito nacional, a proporção de cesarianas também foi superior à de partos vaginais, embora a proporção de tipo de parto ignorado seja elevada em mulheres com HIV no país^7^. Estudos locais também relataram proporção elevada de nascimentos por cesariana em mulheres com HIV^5,27^. A recomendação vigente é de que a cesariana eletiva seja realizada apenas em gestantes com carga viral ignorada ou superior a 1.000 cópias da 34ª semana gestacional em diante^13,28^. Todavia, dados de uma coorte de mulheres com HIV/aids no RJ mostram elevada proporção de nascimentos por cesariana, sem redução no período entre 2000 e 2016^29^, diferentemente de outros países onde foi observado aumento da proporção de partos vaginais em mulheres com HIV^30,31^. Num país com elevada proporção de cesarianas, esses resultados podem refletir tanto o desconhecimento das diretrizes assistenciais, com alargamento das indicações de cesariana em mulheres com HIV como a ausência de exames de carga viral próximas ao parto. Em estudo nacional realizado entre 2011 e 2012^4^, dados sobre exame de carga viral não estavam disponíveis em 45% das gestantes.

O pequeno número de mulheres com HIV limitou a análise dos desfechos gestacionais e maternos, ressaltando-se que todos os desfechos desfavoráveis ocorreram em internações com financiamento público. A proporção de perdas fetais precoces e natimortos foi similar à dos dados nacionais registrados no período entre 2021 e 2023^7^. A estimativa pontual da proporção de baixo peso ao nascer (17,8%) foi elevada e superior à média nacional de 9,4% para o total de NV em 2022, enquanto a estimativa pontual da proporção de prematuros (6,1%) foi inferior à média nacional (11,8%), entretanto o amplo intervalo de confiança não permite afirmar que essas diferenças sejam significativas. Maior prevalência de desfechos perinatais desfavoráveis foi relatada em gestantes com HIV, independentemente do nível de renda e região de residência^32^. A proporção de condições potencialmente ameaçadoras à vida e de *near miss* materno encontrada está em conformidade com os valores estimados para o Brasil^33^, não sendo identificado caso de óbito materno, que é um desfecho mais raro. Maior risco de morbidade materna grave associada à infecção pelo HIV foi relatada em estudos anteriores^34–36^.

Este estudo tem algumas limitações. Não foram incluídas mulheres internadas em hospitais com menos de 100 partos/ano ou com parto não hospitalar, não sendo os resultados aplicáveis a essas mulheres. Na avaliação do PN, a ausência de registro de exames e de tratamento na CG foi considerada como ausência de realização. Embora isso possa ter superestimado a proporção de mulheres não testadas e não tratadas, entendemos que a CG deveria estar adequadamente preenchida. Por fim, não estavam disponíveis dados sobre uso de zidovudina injetável, profilaxia na criança exposta, inibição da lactação e fornecimento de fórmula láctea, nem dados de exames do recém-nascido, limitando a avaliação do manejo hospitalar das mulheres e seus bebês e impedindo o cálculo da TV.

Para concluir, foram identificadas oportunidades perdidas no manejo das gestantes com HIV em hospitalizações públicas e privadas do RJ. A taxa de detecção encontrada foi superior à registrada no SINAN, sugerindo problemas na notificação de casos. Mulheres com financiamento público da atenção hospitalar apresentaram maior vulnerabilidade social e menor acesso à assistência PN e à testagem durante a gestação, sendo todos os desfechos gestacionais e maternos negativos observados nessas mulheres.
